# Supplementary material for: Development of a methodology to make individual estimates of the precision of liquid chromatography-tandem mass spectrometry drug assay results for use in population pharmacokinetic modeling and the optimization of dosage regimens
Source: PLoS One. 2020 Mar 5;15(3):e0229873. doi: 10.1371/journal.pone.0229873 (PMC7058336; doi:10.1371/journal.pone.0229873)
Supplement: S1 Table — CBZ, carbamazepine. FLU, fluconazole. LAM, lamotrigine. LEV, levetiracetam. SS1, Stock solution 1. (DOCX) [file pone.0229873.s001.docx]

| **Calibrator** |  | **CBZ** | **FLU** | **LAM** | **LEV** |
| --- | --- | --- | --- | --- | --- |
| **Experiment 1** | | | | | |
| **Level 6** | **preparation** | **75 µL CBZ-SS1 + 925 µL serum** | **75 µL FLC-SS1 + 925 µL serum** | **75 µL LAM-SS1 + 925 µL serum** | **75 µL LEV-SS1 + 925 µL serum** |
|  | **concentration (µg/mL)** | **301** | **371** | **306** | **298** |
| **Level 5** | **preparation** | **200 µL Level 6 calibrator + 100 µL serum** | | | |
|  | **concentration (µg/mL)** | **201** | **247** | **204** | **199** |
| **Level 4** | **preparation** | **200 µL Level 6 calibrator + 400 µL serum** | | | |
|  | **concentration (µg/mL)** | **100** | **124** | **102** | **99.3** |
| **Level 3** | **preparation** | **30 µL Level 4 calibrator + 470 µL serum** | | | |
|  | **concentration (µg/mL)** | **6.00** | **7.44** | **6.12** | **5.96** |
| **Level 2** | **preparation** | **15 µL Level 4 calibrator + 485 µL serum** | | | |
|  | **concentration (µg/mL)** | **3.00** | **3.72** | **3.06** | **2.98** |
| **Level 1** | **preparation** | **10 µL Level 4 calibrator + 990 µL serum** | | | |
|  | **concentration (µg/mL)** | **1.00** | **1.24** | **1.02** | **1.49** |
| **Experiment 2** | | | | | |
| **Level 6** | **preparation** | **20 µL CBZ-SS2 + 980 µL serum** | **20 µL FLC-SS2 + 980 µL serum** | **20 µL LAM-SS2 + 980 µL serum** | **20 µL LEV-SS2 + 980 µL serum** |
|  | **concentration (µg/mL)** | **40.3** | **40.3** | **39.7** | **40.3** |
| **Level 5** | **preparation** | **12.5 µL CBZ-SS2 + 1000 µL serum** | **12.5 µL FLC-SS2 + 1000 µL serum** | **12.5 µL LAM-SS2 + 1000 µL serum** | **12.5 µL LEV-SS2 + 1000 µL serum** |
|  | **concentration (µg/mL)** | **24.9** | **24.9** | **24.8** | **24.9** |
| **Level 4** | **preparation** | **100 µL Level 6 calibrator + 300 µL serum** | | | |
|  | **concentration (µg/mL)** | **10.1** | **10.1** | **9.93** | **10.1** |
| **Level 3** | **preparation** | **25 µL Level 6 calibrator + 225 µL serum** | | | |
|  | **concentration (µg/mL)** | **4.03** | **4.03** | **3.97** | **4.03** |
| **Level 2** | **preparation** | **25 µL Level 4 calibrator + 225 µL serum** | | | |
|  | **concentration (µg/mL)** | **1.01** | **1.01** | **0.993** | **1.01** |
| **Level 1** | **preparation** | **25 µL Level 3 calibrator + 975 µL serum** | | | |
|  | **concentration (µg/mL)** | **0.403** | **0.403** | **0.397** | **0.403** |
| **Experiment 3** | | | | | |
| **Level 6** | **preparation** | **20 µL CBZ-SS2 + 980 µL serum** | **20 µL FLC-SS2 + 980 µL serum** | **20 µL LAM-SS2 + 980 µL serum** | **49 µL LEV-SS2 + 950 µL serum** |
|  | **concentration (µg/mL)** | **40.3** | **40.3** | **39.7** | **98.8** |
| **Level 5** | **preparation** | **12.5 µL CBZ-SS2 + 1000 µL serum** | **12.5 µL FLC-SS2 + 1000 µL serum** | **12.5 µL LAM-SS2 + 1000 µL serum** | **25 µL LEV-SS2 + 1000 µL serum** |
|  | **concentration (µg/mL)** | **24.9** | **24.9** | **24.8** | **49.2** |
| **Level 4** | **preparation** | **100 µL Level 6 calibrator + 300 µL serum** | | | |
|  | **concentration (µg/mL)** | **10.1** | **10.1** | **9.93** | **24.7** |
| **Level 3** | **preparation** | **25 µL Level 6 calibrator + 225 µL serum** | | | |
|  | **concentration (µg/mL)** | **4.03** | **4.03** | **3.97** | **9.88** |
| **Level 2** | **preparation** | **25 µL Level 4 calibrator + 225 µL serum** | | | |
|  | **concentration (µg/mL)** | **1.01** | **1.01** | **0.993** | **2.47** |
| **Level 1** | **preparation** | **25 µL Level 3 calibrator + 225 µL serum** | | | |
|  | **concentration (µg/mL)** | **0.403** | **0.403** | **0.397** | **0.988** |
