# Supplementary material for: Development of a methodology to make individual estimates of the precision of liquid chromatography-tandem mass spectrometry drug assay results for use in population pharmacokinetic modeling and the optimization of dosage regimens
Source: PLoS One. 2020 Mar 5;15(3):e0229873. doi: 10.1371/journal.pone.0229873 (PMC7058336; doi:10.1371/journal.pone.0229873)
Supplement: S2 Table — CBZ-SS1, carbamazepine stock solution 1. FLU-SS1, fluconazole stock solution 1. LAM-SS1, lamotrigine stock solution 1. LEV-SS1, levetiracetam stock solution 1. CBZ-SS2, carbamazepine stock solution 1. FLU-SS2, fluconazole stock solution 1. LAM-SS2, lamotrigine stock solution 1. LEV-SS2, levetiracetam stock solution 1. (DOCX) [file pone.0229873.s002.docx]

| **Spiking level** |  | **CBZ** | **FLU** | **LAM** | **LEV** |
| --- | --- | --- | --- | --- | --- |

| **Experiment 1** |
| --- |

| **Level 4** | **preparation of samples** | **45 µL CBZ-SS1 + 950 µL serum** | **15 µL FLC-SS1 + 980 µL serum** | **45 µL LAM-SS1 + 950 µL serum** | **38 µL LEV-SS1 + 1000 µL serum** |
| --- | --- | --- | --- | --- | --- |
|  | **concentration (µg/mL)** | **181** | **74.5** | **184** | **150** |
| **Level 3** | **preparation of samples** | **50 µL Level 4 sample + 100 µL serum** | | | |
|  | **concentration (µg/mL)** | **60.3** | **24.8** | **61.4** | **49.9** |
| **Level 2** | **preparation of samples** | **50 µL Level 3 sample + 150 µL serum** | | | |
|  | **concentration (µg/mL)** | **15.1** | **6.21** | **15.4** | **12.5** |
| **Level 1** | **preparation of samples** | **40 µL Level 2 sample + 160 µL serum** | | | |
|  | **concentration (µg/mL)** | **3.02** | **1.24** | **3.07** | **2.49** |

| **Experiment 2** |
| --- |

| **Level 6** | **preparation of samples** | **17 µL CBZ-SS2 + 1000 µL serum** | **8.0 µL FLC-SS2 + 990 µL serum** | **20 µL LAM-SS2 + 970 µL serum** | **16.5 µL LEV-SS2 + 985 µL serum** |
| --- | --- | --- | --- | --- | --- |
|  | **concentration (µg/mL)** | **33.7** | **16.3** | **40.2** | **33.2** |
| **Level 5** | **preparation of samples** | **200 µL Level 6 sample + 200 µL serum** | | | |
|  | **concentration (µg/mL)** | **16.8** | **8.13** | **20.1** | **16.6** |
| **Level 4** | **preparation of samples** | **50 µL Level 6 sample + 150 µL serum** | | | |
|  | **concentration (µg/mL)** | **8.38** | **4.07** | **10.1** | **8.31** |
| **Level 3** | **preparation of samples** | **25 µL Level 6 sample + 375 µL serum** | | | |
|  | **concentration (µg/mL)** | **2.09** | **1.02** | **2.51** | **2.08** |
| **Level 2** | **preparation of samples** | **50 µL Level 3 sample + 200 µL serum** | | | |
|  | **concentration (µg/mL)** | **0.419** | **0.203** | **0.503** | **0.420** |
| **Level 1** | **preparation of samples** | **25 µL Level 3 sample + 225 µL serum** | | | |
|  | **concentration (µg/mL)** | **0.209** | **0.102** | **0.251** | **0.208** |

| **Experiment 3** |
| --- |

| **Level 10** | **preparation of samples** | **6.0 µL CBZ-SS2 + 490 µL serum** | **6.0 µL FLC-SS2 + 490 µL serum** | **6.0 µL LTG-SS2 + 490 µL serum** | **48 µL LEM-SS2 + 950 µL serum** |
| --- | --- | --- | --- | --- | --- |
|  | **concentration (µg/mL)** | **24.3** | **24.3** | **23.9** | **97.1** |
| **Level 9** | **preparation of samples** | **250 µL Level 10 sample + 250 µL serum** | | | |
|  | **concentration (µg/mL)** | **12.2** | **12.2** | **12.0** | **48.5** |
| **Level 8** | **preparation of samples** | **86 µL Level 10 sample + 410 µL serum** | | | |
|  | **concentration (µg/mL)** | **4.21** | **4.21** | **4.14** | **16.8** |
| **Level 7** | **preparation of samples** | **25 µL Level 10 sample + 225 µL serum** | | | |
|  | **concentration (µg/mL)** | **2.43** | **2.43** | **2.39** | **9.71** |
| **Level 6** | **preparation of samples** | **10 µL Level 10 sample + 290 µL serum** | | | |
|  | **concentration (µg/mL)** | **0.810** | **0.810** | **0.797** | **3.24** |
| **Level 5** | **preparation of samples** | **10 µL Level 10 sample + 990 µL serum** | | | |
|  | **concentration (µg/mL)** | **0.243** | **0.243** | **0.239** | **0.971** |
| **Level 4** | **preparation of samples** | **50 µL Level 5 sample + 75 µL serum** | | | |
|  | **concentration (µg/mL)** | **0.0972** | **0.0972** | **0.0956** | **0.388** |
| **Level 3** | **preparation of samples** | **20 µL Level 5 sample + 80 µL serum** | | | |
|  | **concentration (µg/mL)** | **0.0486** | **0.0486** | **0.0478** | **0.194** |
| **Level 2** | **preparation of samples** | **10 µL Level 5 sample + 90 µL serum** | | | |
|  | **concentration (µg/mL)** | **0.0243** | **0.0243** | **0.0239** | **0.0971** |
| **Level 1** | **preparation of samples** | **10 µL Level 5 sample + 190 µL serum** | | | |
|  | **concentration (µg/mL)** | **0.0122** | **0.0122** | **0.0120** | **0.0486** |
