# Supplementary material for: Development of a methodology to make individual estimates of the precision of liquid chromatography-tandem mass spectrometry drug assay results for use in population pharmacokinetic modeling and the optimization of dosage regimens
Source: PLoS One. 2020 Mar 5;15(3):e0229873. doi: 10.1371/journal.pone.0229873 (PMC7058336; doi:10.1371/journal.pone.0229873)
Supplement: S3 Table — (DOCX) [file pone.0229873.s003.docx]

| **A** | **„normal” serum samples** | | | |
| --- | --- | --- | --- | --- |
|  | **carbamazepine** | **fluconazole** | **lamotrigine** | **levetiracetam** |
| ***matrix factors of analytes (%)*** | | | | |
| low level, mean | 102 | 99.0 | 98.1 | 105 |
| low level, CV% | 5.9 | 4.7 | 4.5 | 8.1 |
| high level, mean | 105 | 107 | 99.3 | 97.7 |
| high level, CV% | 3.6 | 3.6 | 3.9 | 4.1 |
| ***internal standard-corrected matrix factors of analytes (%)*** | | | | |
| low level, mean | 106 | 103 | 102 | 102 |
| low level, CV% | 0.44 | 0.25 | 0.35 | 0.33 |
| high level, mean | 112 | 114 | 104 | 103 |
| high level, CV% | 5.7 | 3.8 | 4.7 | 4.0 |
|  | | | | |
| **B** | **hyperbilirubinemic serum samples** | | | |
|  | **carbamazepine** | **fluconazole** | **lamotrigine** | **levetiracetam** |
| ***matrix factors of analytes (%)*** | | | | |
| low level, mean | 105 | 106 | 107 | 105 |
| low level, CV% | 2.5 | 3.9 | 3.7 | 8.3 |
| high level, mean | 110 | 111 | 109 | 110 |
| high level, CV% | 0.8 | 1.0 | 2.4 | 0.9 |
| ***internal standard-corrected matrix factors of analytes (%)*** | | | | |
| low level, mean | 102 | 101 | 99.6 | 102 |
| low level, CV% | 1.4 | 2.1 | 2.3 | 2.2 |
| high level, mean | 100 | 100 | 100 | 101 |
| high level, CV% | 0.4 | 0.5 | 0.5 | 0.5 |
|  | | | | |
| **C** | **hemolytic serum samples** | | | |
|  | **carbamazepine** | **fluconazole** | **lamotrigine** | **levetiracetam** |
| ***matrix factors of analytes (%)*** | | | | |
| low level, mean | 101 | 102 | 108 | 96.7 |
| low level, CV% | 2.8 | 3.0 | 4.4 | 3.4 |
| high level, mean | 109 | 109 | 108 | 108 |
| high level, CV% | 1.4 | 1.7 | 1.4 | 1.3 |
| ***internal standard-corrected matrix factors of analytes (%)*** | | | | |
| low level, mean | 106 | 103 | 102 | 104 |
| low level, CV% | 2.9 | 2.0 | 3.3 | 2.4 |
| high level, mean | 101 | 98.9 | 99.2 | 100 |
| high level, CV% | 1.7 | 1.7 | 1.6 | 1.7 |
|  | | | | |
| **D** | **lipemic serum samples** | | | |
|  | **carbamazepine** | **fluconazole** | **lamotrigine** | **levetiracetam** |
| ***matrix factors of analytes (%)*** | | | | |
| low level, mean | 106 | 106 | 110 | 101 |
| low level, CV% | 1.5 | 1.9 | 1.7 | 1.6 |
| high level, mean | 107 | 108 | 106 | 107 |
| high level, CV% | 3.0 | 2.8 | 3.1 | 2.9 |
| ***internal standard-corrected matrix factors of analytes (%)*** | | | | |
| low level, mean | 105 | 104 | 101 | 103 |
| low level, CV% | 2.9 | 3.8 | 4.1 | 3.9 |
| high level, mean | 100 | 99.5 | 99.4 | 100 |
| high level, CV% | 0.9 | 0.8 | 0.9 | 0.7 |
