# Supplementary material for: Development of a methodology to make individual estimates of the precision of liquid chromatography-tandem mass spectrometry drug assay results for use in population pharmacokinetic modeling and the optimization of dosage regimens
Source: PLoS One. 2020 Mar 5;15(3):e0229873. doi: 10.1371/journal.pone.0229873 (PMC7058336; doi:10.1371/journal.pone.0229873)
Supplement: S4 Table — OLS, unweighted linear least squares. WLS, 1/x2-weighted linear least squares. (DOCX) [file pone.0229873.s004.docx]

| **level** | **nominal concentration (µg/mL)** | **observed standard deviation (µg/mL)** | **predicted/observed standard deviation (%)** | | | | | |
| --- | --- | --- | --- | --- | --- | --- | --- | --- |
|  |  |  | **Theil** | **Theil-Siegel** | **WLS** | **OLS** | **2^nd^-order polynomial** | **3^rd^-order polynomial** |
| 1 | 0.000 | 0.008 | 17.1 | 17.5 | 19.8 | -1224 | -504 | 403 |
| 2 | 0.012 | 0.002 | 100 | 102 | 100 | -4760 | -1940 | 1600 |
| 3 | 0.024 | 0.002 | 143 | 145 | 132 | -5047 | -2037 | 1730 |
| 4 | 0.050 | 0.004 | 102 | 103 | 85.6 | -2309 | -913 | 825 |
| 5 | 0.097 | 0.007 | 95.2 | 95.6 | 74.3 | -1276 | -484 | 492 |
| 6 | 0.209 | 0.018 | 74.4 | 74.6 | 54.7 | -477 | -160 | 220 |
| 7 | 0.243 | 0.011 | 144.3 | 144.6 | 105.2 | -786 | -253 | 382 |
| 8 | 0.419 | 0.041 | 62.9 | 63.0 | 44.8 | -177 | -40.7 | 114 |
| 9 | 0.810 | 0.020 | 239 | 240 | 168 | -225 | 32.9 | 294 |
| 10 | 2.09 | 0.140 | 88.0 | 88.0 | 60.8 | 30.4 | 59.6 | 73.8 |
| 11 | 2.43 | 0.062 | 230 | 230 | 159 | 106 | 167 | 186 |
| 12 | 3.02 | 0.275 | 64.6 | 64.7 | 44.6 | 38.9 | 50.8 | 49.8 |
| 13 | 4.21 | 0.156 | 158 | 158 | 109 | 121 | 135 | 116 |
| 14 | 8.38 | 0.458 | 107 | 107 | 73.7 | 104 | 101 | 77.6 |
| 15 | 12.2 | 0.440 | 162 | 162 | 111 | 169 | 158 | 122 |
| 16 | 15.1 | 0.983 | 89.9 | 89.9 | 61.7 | 95.9 | 89.1 | 70.0 |
| 17 | 16.8 | 0.995 | 98.8 | 98.8 | 67.9 | 107 | 98.6 | 78.7 |
| 18 | 24.3 | 0.987 | 144 | 144 | 98.9 | 160 | 147 | 126 |
| 19 | 33.5 | 1.71 | 115 | 115 | 78.8 | 130 | 119 | 111 |
| 20 | 60.3 | 4.26 | 82.7 | 82.7 | 56.8 | 95.6 | 88.6 | 98.2 |
| 21 | 181 | 12.6 | 84.2 | 84.2 | 57.8 | 99.0 | 100 | 100 |

**OLS, unweighted linear least squares. WLS, 1/x^2^-weighted linear least squares.**
