# Supplementary material for: Development of a methodology to make individual estimates of the precision of liquid chromatography-tandem mass spectrometry drug assay results for use in population pharmacokinetic modeling and the optimization of dosage regimens
Source: PLoS One. 2020 Mar 5;15(3):e0229873. doi: 10.1371/journal.pone.0229873 (PMC7058336; doi:10.1371/journal.pone.0229873)
Supplement: S5 Table — OLS, unweighted linear least squares. WLS, 1/x2-weighted linear least squares. (DOCX) [file pone.0229873.s005.docx]

| **level** | **nominal concentration (µg/mL)** | **observed standard deviation (µg/mL)** | **predicted/observed standard deviation (%)** | | | | | |
| --- | --- | --- | --- | --- | --- | --- | --- | --- |
|  |  |  | **Theil** | **Theil-Siegel** | **WLS** | **OLS** | **2^nd^-order polynomial** | **3^rd^-order polynomial** |
| 1 | 0.00 | 0.006 | 52.2 | 65.5 | 29.1 | 141 | -184 | 232 |
| 2 | 0.012 | 0.002 | 184 | 224 | 112 | 448 | -516 | 708 |
| 3 | 0.024 | 0.002 | 200 | 238 | 128 | 448 | -456 | 685 |
| 4 | 0.049 | 0.004 | 150 | 171 | 103 | 293 | -229 | 421 |
| 5 | 0.097 | 0.008 | 100 | 110 | 73.8 | 167 | -74.4 | 218 |
| 6 | 0.102 | 0.011 | 74.2 | 81.5 | 55.0 | 123 | -51.0 | 159 |
| 7 | 0.203 | 0.026 | 49.1 | 52.2 | 38.6 | 69.0 | -0.450 | 78.2 |
| 8 | 0.243 | 0.011 | 139 | 146 | 110 | 187 | 18.9 | 205 |
| 9 | 0.810 | 0.024 | 177 | 180 | 148 | 198 | 135 | 173 |
| 10 | 1.02 | 0.070 | 74.8 | 75.9 | 62.8 | 81.8 | 62.2 | 69.6 |
| 11 | 1.24 | 0.077 | 82.3 | 83.3 | 69.3 | 88.6 | 72.3 | 73.8 |
| 12 | 2.43 | 0.089 | 136 | 137 | 116 | 142 | 135 | 114 |
| 13 | 4.07 | 0.155 | 130 | 130 | 111 | 132 | 133 | 108 |
| 14 | 4.21 | 0.220 | 94.3 | 94.7 | 80.5 | 96.0 | 97.4 | 78.7 |
| 15 | 6.21 | 0.342 | 88.8 | 89.0 | 75.9 | 89.7 | 93.3 | 76.2 |
| 16 | 8.13 | 0.434 | 91.5 | 91.7 | 78.3 | 92.1 | 96.9 | 81.2 |
| 17 | 12.2 | 0.362 | 164 | 164 | 141 | 165 | 175 | 156 |
| 18 | 16.3 | 0.814 | 97.4 | 97.5 | 83.4 | 97.3 | 104 | 98.7 |
| 19 | 24.3 | 1.78 | 66.3 | 66.3 | 56.8 | 66.1 | 69.9 | 73.7 |
| 20 | 24.8 | 0.928 | 130 | 130 | 111 | 130 | 137 | 145 |
| 21 | 74.5 | 3.52 | 103 | 103 | 88.1 | 102 | 100 | 100 |

**OLS, unweighted linear least squares. WLS, 1/x^2^-weighted linear least squares.**
