# Supplementary material for: Development of a methodology to make individual estimates of the precision of liquid chromatography-tandem mass spectrometry drug assay results for use in population pharmacokinetic modeling and the optimization of dosage regimens
Source: PLoS One. 2020 Mar 5;15(3):e0229873. doi: 10.1371/journal.pone.0229873 (PMC7058336; doi:10.1371/journal.pone.0229873)
Supplement: S6 Table — OLS, unweighted linear least squares. WLS, 1/x2-weighted linear least squares. (DOCX) [file pone.0229873.s006.docx]

| **level** | **nominal concentration (µg/mL)** | **observed standard deviation (µg/mL)** | **predicted/observed standard deviation (%)** | | | | | |
| --- | --- | --- | --- | --- | --- | --- | --- | --- |
|  |  |  | **Theil** | **Theil-Siegel** | **WLS** | **OLS** | **2^nd^-order polynomial** | **3^rd^-order polynomial** |
| 1 | 0.00 | 0.005 | 31.4 | 46.1 | 46.2 | -422 | -2282 | 1313 |
| 2 | 0.0120 | 0.002 | 101 | 136 | 129 | -969 | -5372 | 3115 |
| 3 | 0.0239 | 0.003 | 100 | 127 | 116 | -734 | -4178 | 2442 |
| 4 | 0.0478 | 0.005 | 76.3 | 91.0 | 79.0 | -369 | -2219 | 1318 |
| 5 | 0.0956 | 0.008 | 70.2 | 78.7 | 64.7 | -183 | -1246 | 765 |
| 6 | 0.239 | 0.009 | 134 | 141 | 110 | -81 | -1025 | 699 |
| 7 | 0.251 | 0.017 | 75.1 | 79.2 | 61.3 | -39.9 | -547 | 376 |
| 8 | 0.503 | 0.043 | 55.7 | 57.4 | 43.2 | 15.7 | -176 | 151 |
| 9 | 0.800 | 0.023 | 163 | 166 | 124 | 99.3 | -245 | 291 |
| 10 | 2.39 | 0.054 | 203 | 204 | 150 | 202 | 92.6 | 148 |
| 11 | 2.51 | 0.107 | 106 | 107 | 78.5 | 107 | 53.7 | 75.3 |
| 12 | 3.07 | 0.217 | 64.0 | 64.3 | 47.2 | 66.7 | 43.6 | 40.3 |
| 13 | 4.14 | 0.103 | 181 | 182 | 133 | 196 | 160 | 100 |
| 14 | 10 | 0.532 | 85.4 | 85.5 | 62.5 | 98.4 | 105 | 47.1 |
| 15 | 12 | 0.263 | 205 | 205 | 150 | 238 | 259 | 121 |
| 16 | 15 | 0.893 | 77.5 | 77.6 | 56.6 | 90.6 | 101 | 52.0 |
| 17 | 20.1 | 0.897 | 101 | 101 | 73.5 | 118 | 134 | 79.6 |
| 18 | 23.9 | 0.819 | 131 | 131 | 95.7 | 155 | 176 | 116 |
| 19 | 40.2 | 1.65 | 109 | 109 | 79.7 | 130 | 149 | 139 |
| 20 | 61.4 | 4.89 | 56.3 | 56.3 | 41.1 | 67.2 | 75.8 | 94.2 |
| 21 | 184 | 9.55 | 86.4 | 86.4 | 63.0 | 104 | 101 | 100 |

**OLS, unweighted linear least squares. WLS, 1/x^2^-weighted linear least squares.**
