# Supplementary material for: Development of a methodology to make individual estimates of the precision of liquid chromatography-tandem mass spectrometry drug assay results for use in population pharmacokinetic modeling and the optimization of dosage regimens
Source: PLoS One. 2020 Mar 5;15(3):e0229873. doi: 10.1371/journal.pone.0229873 (PMC7058336; doi:10.1371/journal.pone.0229873)
Supplement: S7 Table — OLS, unweighted linear least squares. WLS, 1/x2-weighted linear least squares. (DOCX) [file pone.0229873.s007.docx]

| **level** | **nominal concentration (µg/mL)** | **observed standard deviation (µg/mL)** | **predicted/observed standard deviation (%)** | | | | | |
| --- | --- | --- | --- | --- | --- | --- | --- | --- |
|  |  |  | **Theil** | **Theil-Siegel** | **WLS** | **OLS** | **2^nd^-order polynomial** | **3^rd^-order polynomial** |
| 1 | 0.00 | 0.007 | 61.5 | 79.4 | 88.4 | -2078 | -154 | 942 |
| 2 | 0.0486 | 0.006 | 121 | 142 | 134 | -2467 | -151 | 1157 |
| 3 | 0.0971 | 0.008 | 113 | 128 | 110 | -1649 | -79.7 | 800 |
| 4 | 0.194 | 0.014 | 100 | 108 | 83.1 | -900 | -18.5 | 468 |
| 5 | 0.208 | 0.017 | 86.0 | 92.8 | 70.4 | -731 | -11.9 | 384 |
| 6 | 0.388 | 0.033 | 74.0 | 77.6 | 53.9 | -356 | 15.3 | 213 |
| 7 | 0.416 | 0.028 | 93.3 | 97.6 | 67.2 | -417 | 22.2 | 256 |
| 8 | 0.971 | 0.037 | 148 | 151 | 96 | -226 | 75.9 | 219 |
| 9 | 2.08 | 0.134 | 83.7 | 84.6 | 51.5 | -15.0 | 53.8 | 76.9 |
| 10 | 2.49 | 0.205 | 65.3 | 65.8 | 39.8 | 1.62 | 43.3 | 54.6 |
| 11 | 3.24 | 0.072 | 239 | 241 | 145 | 64 | 165 | 177 |
| 12 | 8.31 | 0.402 | 108 | 109 | 64.2 | 83.4 | 81.1 | 63.3 |
| 13 | 9.71 | 0.255 | 199 | 200 | 118 | 163 | 151 | 116 |
| 14 | 12.5 | 0.732 | 89.4 | 89.6 | 52.7 | 78.6 | 68.8 | 52.3 |
| 15 | 16.6 | 0.379 | 229 | 229 | 135 | 214 | 180 | 139 |
| 16 | 16.8 | 0.730 | 120 | 120 | 70.7 | 112 | 94.5 | 73.0 |
| 17 | 33.2 | 1.44 | 120 | 120 | 70.2 | 122 | 100 | 88.0 |
| 18 | 48.5 | 0.867 | 291 | 292 | 171 | 304 | 256 | 248 |
| 19 | 49.9 | 3.06 | 84.8 | 84.8 | 49.7 | 88.5 | 74.9 | 73.1 |
| 20 | 97.1 | 5.71 | 88.5 | 88.5 | 51.8 | 94.7 | 89.5 | 97.6 |
| 21 | 150 | 8.80 | 88.7 | 88.7 | 51.9 | 95.8 | 102 | 100 |
