# Supplementary material for: Development of a methodology to make individual estimates of the precision of liquid chromatography-tandem mass spectrometry drug assay results for use in population pharmacokinetic modeling and the optimization of dosage regimens
Source: PLoS One. 2020 Mar 5;15(3):e0229873. doi: 10.1371/journal.pone.0229873 (PMC7058336; doi:10.1371/journal.pone.0229873)
Supplement: S3 File — (DOCX) [file pone.0229873.s010.docx]

**Script 1: combinatorial calculation of regression coefficients, unweighted linear least squares regression, 4 spiking levels**

print("Select files containing drug levels and assay results")

aepconc=read.csv(file.choose())

aepresults=read.csv(file.choose())

l0=subset(aepresults,aepresults$lvl==0)

l1=subset(aepresults,aepresults$lvl==1)

l2=subset(aepresults,aepresults$lvl==2)

l3=subset(aepresults,aepresults$lvl==3)

l4=subset(aepresults,aepresults$lvl==4)

sd.l0=NULL

sd.l1=NULL

sd.l2=NULL

sd.l3=NULL

sd.l4=NULL

int=NULL

sl=NULL

rsq=NULL

absc=aepconc[colnames(aepconc)==colnames(aepresults)[[4]]]

j=20

index=combn(nrow(l0),j)

comb.l0=matrix(l0[index,4],nrow=nrow(index), ncol=ncol(index))

comb.l1=matrix(l1[index,4],nrow=nrow(index), ncol=ncol(index))

comb.l2=matrix(l2[index,4],nrow=nrow(index), ncol=ncol(index))

comb.l3=matrix(l3[index,4],nrow=nrow(index), ncol=ncol(index))

comb.l4=matrix(l4[index,4],nrow=nrow(index), ncol=ncol(index))

sd.l0=apply(comb.l0,2,sd,na.rm=TRUE)

sd.l1=apply(comb.l1,2,sd,na.rm=TRUE)

sd.l2=apply(comb.l2,2,sd,na.rm=TRUE)

sd.l3=apply(comb.l3,2,sd,na.rm=TRUE)

sd.l4=apply(comb.l4,2,sd,na.rm=TRUE)

sd.l=rbind(sd.l0,sd.l1,sd.l2,sd.l3,sd.l4)

rm(comb.l0)

rm(comb.l1)

rm(comb.l2)

rm(comb.l3)

rm(comb.l4)

rm(sd.l0)

rm(sd.l1)

rm(sd.l2)

rm(sd.l3)

rm(sd.l4)

sumy=colSums(sd.l)

ysquare=colSums(sd.l^2)

absc.df=rep(absc,ncol(sd.l))

absc.df=as.data.frame(absc.df)

prod.sdl=absc.df*sd.l

sumprod.sdl=colSums(prod.sdl)

sl=(nrow(absc)*sumprod.sdl-sum(absc[,1])*sumy)/(nrow(absc)*sum(absc^2)-(sum(absc))^2)

int=(sumy-sl*sum(absc))/nrow(absc)

rsd=apply(sd.l,2,sd)

sd.absc=sd(absc[,1])

sdx=rep(sd.absc,length(rsd))

sdmeans=colMeans(sd.l)

sdmeans.m=t(matrix(rep(sdmeans,nrow(sd.l)),ncol=nrow(sd.l)))

dify=sd.l-sdmeans.m

dify=as.data.frame(dify)

difx=absc-colMeans(absc)

difx.df=rep(difx,ncol(sd.l))

ncoldifx.df=as.data.frame(difx.df)

proddif=difx.df*dify

sdcov=colSums(proddif)/(nrow(absc)-1)

rsq=(sdcov/(sdx*rsd))^2

nni=length(subset(int,int>=0))/length(int)

**Script 2: combinatorial calculation of regression coefficients, ordinary 2^nd^-order least squares regression, 4 spiking levels**

print("Select files containing drug levels and assay results")

aepconc=read.csv(file.choose())

aepresults=read.csv(file.choose())

l0=subset(aepresults,aepresults$lvl==0)

l1=subset(aepresults,aepresults$lvl==1)

l2=subset(aepresults,aepresults$lvl==2)

l3=subset(aepresults,aepresults$lvl==3)

l4=subset(aepresults,aepresults$lvl==4)

sd.l0=NULL

sd.l1=NULL

sd.l2=NULL

sd.l3=NULL

sd.l4=NULL

int=NULL

sl1=NULL

sl2=NULL

rsq=NULL

absc=aepconc[colnames(aepconc)==colnames(aepresults)[[4]]]

indep=absc[,1]

j=6

index=combn(nrow(l0),j)

for (i in 1:ncol(index)) {

sd.l0[i]=sd(subset(l0[l0$sample[index[,i]],4],l0[l0$sample[index[,i]],4]!="NA"))

sd.l1[i]=sd(subset(l1[l1$sample[index[,i]],4],l1[l1$sample[index[,i]],4]!="NA"))

sd.l2[i]=sd(subset(l2[l2$sample[index[,i]],4],l2[l2$sample[index[,i]],4]!="NA"))

sd.l3[i]=sd(subset(l3[l3$sample[index[,i]],4],l3[l3$sample[index[,i]],4]!="NA"))

sd.l4[i]=sd(subset(l4[l4$sample[index[,i]],4],l4[l4$sample[index[,i]],4]!="NA"))

ord=c(sd.l0[i],sd.l1[i],sd.l2[i],sd.l3[i],sd.l4[i])

reg=lm(ord~poly(indep,2,raw=TRUE))

sl1[i]=reg$coefficients[[2]]

sl2[i]=reg$coefficients[[3]]

int[i]=reg$coefficients[[1]]

rsq[i]=summary(reg)$r.squared

}

nni=length(subset(int,int>=0))/length(int)

**Script 3: combinatorial calculation of regression coefficients, ordinary 3^rd^-order least squares regression, 4 spiking levels**

print("Select files containing drug levels and assay results")

aepconc=read.csv(file.choose())

aepresults=read.csv(file.choose())

l0=subset(aepresults,aepresults$lvl==0)

l1=subset(aepresults,aepresults$lvl==1)

l2=subset(aepresults,aepresults$lvl==2)

l3=subset(aepresults,aepresults$lvl==3)

l4=subset(aepresults,aepresults$lvl==4)

sd.l0=NULL

sd.l1=NULL

sd.l2=NULL

sd.l3=NULL

sd.l4=NULL

int=NULL

sl1=NULL

sl2=NULL

sl3=NULL

rsq=NULL

absc=aepconc[colnames(aepconc)==colnames(aepresults)[[4]]]

indep=absc[,1]

j=6

index=combn(nrow(l0),j)

for (i in 1:ncol(index)) {

sd.l0[i]=sd(subset(l0[l0$sample[index[,i]],4],l0[l0$sample[index[,i]],4]!="NA"))

sd.l1[i]=sd(subset(l1[l1$sample[index[,i]],4],l1[l1$sample[index[,i]],4]!="NA"))

sd.l2[i]=sd(subset(l2[l2$sample[index[,i]],4],l2[l2$sample[index[,i]],4]!="NA"))

sd.l3[i]=sd(subset(l3[l3$sample[index[,i]],4],l3[l3$sample[index[,i]],4]!="NA"))

sd.l4[i]=sd(subset(l4[l4$sample[index[,i]],4],l4[l4$sample[index[,i]],4]!="NA"))

ord=c(sd.l0[i],sd.l1[i],sd.l2[i],sd.l3[i],sd.l4[i])

reg=lm(ord~poly(indep,3,raw=TRUE))

sl1[i]=reg$coefficients[[2]]

sl2[i]=reg$coefficients[[3]]

sl3[i]=reg$coefficients[[4]]

int[i]=reg$coefficients[[1]]

rsq[i]=summary(reg)$r.squared

}

**Script 4: combinatorial calculation of regression coefficients, 1/x^2^-weighted linear least squares regression, 4 spiking levels**

print("Select files containing drug levels and assay results")

aepconc=read.csv(file.choose())

aepresults=read.csv(file.choose())

aepconc[1,2:ncol(aepconc)]=0.0000001

l0=subset(aepresults,aepresults$lvl==0)

l1=subset(aepresults,aepresults$lvl==1)

l2=subset(aepresults,aepresults$lvl==2)

l3=subset(aepresults,aepresults$lvl==3)

l4=subset(aepresults,aepresults$lvl==4)

sd.l0=NULL

sd.l1=NULL

sd.l2=NULL

sd.l3=NULL

sd.l4=NULL

int=NULL

sl=NULL

absc=aepconc[colnames(aepconc)==colnames(aepresults)[[4]]]

j=20

wt=1/absc[,1]^2

index=combn(nrow(l0),j)

comb.l0=matrix(l0[index,4],nrow=nrow(index), ncol=ncol(index))

comb.l1=matrix(l1[index,4],nrow=nrow(index), ncol=ncol(index))

comb.l2=matrix(l2[index,4],nrow=nrow(index), ncol=ncol(index))

comb.l3=matrix(l3[index,4],nrow=nrow(index), ncol=ncol(index))

comb.l4=matrix(l4[index,4],nrow=nrow(index), ncol=ncol(index))

sd.l0=apply(comb.l0,2,sd,na.rm=TRUE)

sd.l1=apply(comb.l1,2,sd,na.rm=TRUE)

sd.l2=apply(comb.l2,2,sd,na.rm=TRUE)

sd.l3=apply(comb.l3,2,sd,na.rm=TRUE)

sd.l4=apply(comb.l4,2,sd,na.rm=TRUE)

sd.l=rbind(sd.l0,sd.l1,sd.l2,sd.l3,sd.l4)

wt.df=rep(wt,ncol(sd.l))

wt.df=matrix(wt.df,nrow=length(absc[,1]))

wt.df=as.data.frame(wt.df)

wtsd.l=wt.df*sd.l

rm(comb.l0)

rm(comb.l1)

rm(comb.l2)

rm(comb.l3)

rm(comb.l4)

rm(sd.l0)

rm(sd.l1)

rm(sd.l2)

rm(sd.l3)

rm(sd.l4)

sumwt=sum(wt)

sumx=sum(wt*absc[,1])

sumy=colSums(wtsd.l)

xsquare=sum(wt*absc[,1]^2)

ysquare=colSums(wtsd.l^2)

wtx=wt*absc[,1]

prod.sdl=absc[,1]*wtsd.l

sumprod.sdl=colSums(prod.sdl)

sl=(sumwt*sumprod.sdl-sumx*sumy)/(sumwt*xsquare-sumx^2)

int=(sumy-sl*sumx)/sumwt

meanxw=sumx/sumwt

meanyw=sumy/sumwt

meanyw=rep(meanyw,length(absc[,1]))

meanyw=matrix(meanyw,nrow=length(absc[,1]),byrow=T)

difx=wt*(absc[,1]-meanxw)

wtdifx=rep(difx,ncol(sd.l))

wtdifmat=matrix(wtdifx,nrow=length(absc[,1]))

wtdifmat=as.data.frame(wtdifmat)

dify=sd.l-meanyw

covxyw=colSums(wtdifmat*dify)/sumwt

covxxw=sum(difx*(absc[,1]-meanxw))/sumwt

covxxw=rep(covxxw,ncol(sd.l))

covyyw=colSums(wt*dify^2)/sumwt

rsq=covxyw^2/(covxxw*covyyw)

nni=length(subset(int,int>=0))/length(int)

**Script 5: combinatorial calculation of regression coefficients, Theil’s regression (with or without the Siegel estimator), 4 spiking levels**

print("Select files containing drug levels and assay results")

aepconc=read.csv(file.choose())

aepresults=read.csv(file.choose())

l0=subset(aepresults,aepresults$lvl==0)

l1=subset(aepresults,aepresults$lvl==1)

l2=subset(aepresults,aepresults$lvl==2)

l3=subset(aepresults,aepresults$lvl==3)

l4=subset(aepresults,aepresults$lvl==4)

sd.l0=NULL

sd.l1=NULL

sd.l2=NULL

sd.l3=NULL

sd.l4=NULL

int=NULL

sl=NULL

absc=aepconc[colnames(aepconc)==colnames(aepresults)[[4]]]

indep=absc[,1]

j=20

index=combn(nrow(l0),j)

for (i in 1:ncol(index)) {

sd.l0[i]=sd(subset(l0[l0$sample[index[,i]],4],l0[l0$sample[index[,i]],4]!="NA"))

sd.l1[i]=sd(subset(l1[l1$sample[index[,i]],4],l1[l1$sample[index[,i]],4]!="NA"))

sd.l2[i]=sd(subset(l2[l2$sample[index[,i]],4],l2[l2$sample[index[,i]],4]!="NA"))

sd.l3[i]=sd(subset(l3[l3$sample[index[,i]],4],l3[l3$sample[index[,i]],4]!="NA"))

sd.l4[i]=sd(subset(l4[l4$sample[index[,i]],4],l4[l4$sample[index[,i]],4]!="NA"))

ord=c(sd.l0[i],sd.l1[i],sd.l2[i],sd.l3[i],sd.l4[i])

reg=mblm(ord~indep, repeated=FALSE)

sl[i]=summary(reg)$coefficients[[2]]

int[i]=summary(reg)$coefficients[[1]]

}

nni=length(subset(int,int>=0))/length(int)

**Script 6: combinatorial calculation of regression coefficients, unweighted linear least squares regression, 6 spiking levels**

print("Select files containing drug levels and assay results")

aepconc=read.csv(file.choose())

aepresults=read.csv(file.choose())

l0=subset(aepresults,aepresults$lvl==0)

l1=subset(aepresults,aepresults$lvl==1)

l2=subset(aepresults,aepresults$lvl==2)

l3=subset(aepresults,aepresults$lvl==3)

l4=subset(aepresults,aepresults$lvl==4)

l5=subset(aepresults,aepresults$lvl==5)

l6=subset(aepresults,aepresults$lvl==6)

sd.l0=NULL

sd.l1=NULL

sd.l2=NULL

sd.l3=NULL

sd.l4=NULL

sd.l5=NULL

sd.l6=NULL

int=NULL

sl=NULL

rsq=NULL

absc=aepconc[colnames(aepconc)==colnames(aepresults)[[4]]]

j=6

index=combn(nrow(l0),j)

comb.l0=matrix(l0[index,4],nrow=nrow(index), ncol=ncol(index))

comb.l1=matrix(l1[index,4],nrow=nrow(index), ncol=ncol(index))

comb.l2=matrix(l2[index,4],nrow=nrow(index), ncol=ncol(index))

comb.l3=matrix(l3[index,4],nrow=nrow(index), ncol=ncol(index))

comb.l4=matrix(l4[index,4],nrow=nrow(index), ncol=ncol(index))

comb.l5=matrix(l5[index,4],nrow=nrow(index), ncol=ncol(index))

comb.l6=matrix(l6[index,4],nrow=nrow(index), ncol=ncol(index))

sd.l0=apply(comb.l0,2,sd,na.rm=TRUE)

sd.l1=apply(comb.l1,2,sd,na.rm=TRUE)

sd.l2=apply(comb.l2,2,sd,na.rm=TRUE)

sd.l3=apply(comb.l3,2,sd,na.rm=TRUE)

sd.l4=apply(comb.l4,2,sd,na.rm=TRUE)

sd.l5=apply(comb.l5,2,sd,na.rm=TRUE)

sd.l6=apply(comb.l6,2,sd,na.rm=TRUE)

sd.l=rbind(sd.l0,sd.l1,sd.l2,sd.l3,sd.l4,sd.l5,sd.l6)

rm(comb.l0)

rm(comb.l1)

rm(comb.l2)

rm(comb.l3)

rm(comb.l4)

rm(comb.l5)

rm(comb.l6)

rm(sd.l0)

rm(sd.l1)

rm(sd.l2)

rm(sd.l3)

rm(sd.l4)

rm(sd.l5)

rm(sd.l6)

sumy=colSums(sd.l)

ysquare=colSums(sd.l^2)

absc.df=rep(absc,ncol(sd.l))

absc.df=as.data.frame(absc.df)

prod.sdl=absc.df*sd.l

sumprod.sdl=colSums(prod.sdl)

sl=(nrow(absc)*sumprod.sdl-sum(absc[,1])*sumy)/(nrow(absc)*sum(absc^2)-(sum(absc))^2)

int=(sumy-sl*sum(absc))/nrow(absc)

rsd=apply(sd.l,2,sd)

sd.absc=sd(absc[,1])

sdx=rep(sd.absc,length(rsd))

sdmeans.m=t(matrix(rep(sdmeans,nrow(sd.l)),ncol=nrow(sd.l)))

dify=sd.l-sdmeans.m

dify=as.data.frame(dify)

difx=absc-colMeans(absc)

difx.df=rep(difx,ncol(sd.l))

ncoldifx.df=as.data.frame(difx.df)

proddif=difx.df*dify

sdcov=colSums(proddif)/(nrow(absc)-1)

rsq=(sdcov/(sdx*rsd))^2

nni=length(subset(int,int>=0))/length(int)

**Script 7: combinatorial calculation of regression coefficients, ordinary 2^nd^-order least squares regression, 6 spiking levels**

print("Select files containing drug levels and assay results")

aepconc=read.csv(file.choose())

aepresults=read.csv(file.choose())

l0=subset(aepresults,aepresults$lvl==0)

l1=subset(aepresults,aepresults$lvl==1)

l2=subset(aepresults,aepresults$lvl==2)

l3=subset(aepresults,aepresults$lvl==3)

l4=subset(aepresults,aepresults$lvl==4)

l5=subset(aepresults,aepresults$lvl==5)

l6=subset(aepresults,aepresults$lvl==6)

sd.l0=NULL

sd.l1=NULL

sd.l2=NULL

sd.l3=NULL

sd.l4=NULL

sd.l5=NULL

sd.l6=NULL

int=NULL

sl1=NULL

sl2=NULL

rsq=NULL

absc=aepconc[colnames(aepconc)==colnames(aepresults)[[4]]]

indep=absc[,1]

j=20

index=combn(nrow(l0),j)

for (i in 1:ncol(index)) {

sd.l0[i]=sd(subset(l0[l0$sample[index[,i]],4],l0[l0$sample[index[,i]],4]!="NA"))

sd.l1[i]=sd(subset(l1[l1$sample[index[,i]],4],l1[l1$sample[index[,i]],4]!="NA"))

sd.l2[i]=sd(subset(l2[l2$sample[index[,i]],4],l2[l2$sample[index[,i]],4]!="NA"))

sd.l3[i]=sd(subset(l3[l3$sample[index[,i]],4],l3[l3$sample[index[,i]],4]!="NA"))

sd.l4[i]=sd(subset(l4[l4$sample[index[,i]],4],l4[l4$sample[index[,i]],4]!="NA"))

sd.l5[i]=sd(subset(l5[l5$sample[index[,i]],4],l5[l5$sample[index[,i]],4]!="NA"))

sd.l6[i]=sd(subset(l6[l6$sample[index[,i]],4],l6[l6$sample[index[,i]],4]!="NA"))

ord=c(sd.l0[i],sd.l1[i],sd.l2[i],sd.l3[i],sd.l4[i],sd.l5[i],sd.l6[i])

reg=lm(ord~poly(indep,2,raw=TRUE))

sl1[i]=reg$coefficients[[2]]

sl2[i]=reg$coefficients[[3]]

int[i]=reg$coefficients[[1]]

rsq[i]=summary(reg)$r.squared

}

nni=length(subset(int,int>=0))/length(int)

**Script 8: combinatorial calculation of regression coefficients, unweighted 3^rd^-order least squares regression, 6 spiking levels**

print("Select files containing drug levels and assay results")

aepconc=read.csv(file.choose())

aepresults=read.csv(file.choose())

l0=subset(aepresults,aepresults$lvl==0)

l1=subset(aepresults,aepresults$lvl==1)

l2=subset(aepresults,aepresults$lvl==2)

l3=subset(aepresults,aepresults$lvl==3)

l4=subset(aepresults,aepresults$lvl==4)

l5=subset(aepresults,aepresults$lvl==5)

l6=subset(aepresults,aepresults$lvl==6)

sd.l0=NULL

sd.l1=NULL

sd.l2=NULL

sd.l3=NULL

sd.l4=NULL

sd.l5=NULL

sd.l6=NULL

int=NULL

sl1=NULL

sl2=NULL

sl3=NULL

rsq=NULL

absc=aepconc[colnames(aepconc)==colnames(aepresults)[[4]]]

indep=absc[,1]

j=6

index=combn(nrow(l0),j)

for (i in 1:ncol(index)) {

sd.l0[i]=sd(subset(l0[l0$sample[index[,i]],4],l0[l0$sample[index[,i]],4]!="NA"))

sd.l1[i]=sd(subset(l1[l1$sample[index[,i]],4],l1[l1$sample[index[,i]],4]!="NA"))

sd.l2[i]=sd(subset(l2[l2$sample[index[,i]],4],l2[l2$sample[index[,i]],4]!="NA"))

sd.l3[i]=sd(subset(l3[l3$sample[index[,i]],4],l3[l3$sample[index[,i]],4]!="NA"))

sd.l4[i]=sd(subset(l4[l4$sample[index[,i]],4],l4[l4$sample[index[,i]],4]!="NA"))

sd.l5[i]=sd(subset(l5[l5$sample[index[,i]],4],l5[l5$sample[index[,i]],4]!="NA"))

sd.l6[i]=sd(subset(l6[l6$sample[index[,i]],4],l6[l6$sample[index[,i]],4]!="NA"))

ord=c(sd.l0[i],sd.l1[i],sd.l2[i],sd.l3[i],sd.l4[i],sd.l5[i],sd.l6[i])

reg=lm(ord~poly(indep,3,raw=TRUE))

sl1[i]=reg$coefficients[[2]]

sl2[i]=reg$coefficients[[3]]

sl3[i]=reg$coefficients[[4]]

int[i]=reg$coefficients[[1]]

rsq[i]=summary(reg)$r.squared

}

nni=length(subset(int,int>=0))/length(int)

**Script 9: combinatorial calculation of regression coefficients, 1/x^2^-weighted linear least squares regression, 6 spiking levels**

print("Select files containing drug levels and assay results")

aepconc=read.csv(file.choose())

aepresults=read.csv(file.choose())

aepconc[1,2:ncol(aepconc)]=0.0000001

l0=subset(aepresults,aepresults$lvl==0)

l1=subset(aepresults,aepresults$lvl==1)

l2=subset(aepresults,aepresults$lvl==2)

l3=subset(aepresults,aepresults$lvl==3)

l4=subset(aepresults,aepresults$lvl==4)

l5=subset(aepresults,aepresults$lvl==5)

l6=subset(aepresults,aepresults$lvl==6)

sd.l0=NULL

sd.l1=NULL

sd.l2=NULL

sd.l3=NULL

sd.l4=NULL

sd.l5=NULL

sd.l6=NULL

int=NULL

sl=NULL

absc=aepconc[colnames(aepconc)==colnames(aepresults)[[4]]]

j=20

wt=1/absc[,1]^2

index=combn(nrow(l0),j)

comb.l0=matrix(l0[index,4],nrow=nrow(index), ncol=ncol(index))

comb.l1=matrix(l1[index,4],nrow=nrow(index), ncol=ncol(index))

comb.l2=matrix(l2[index,4],nrow=nrow(index), ncol=ncol(index))

comb.l3=matrix(l3[index,4],nrow=nrow(index), ncol=ncol(index))

comb.l4=matrix(l4[index,4],nrow=nrow(index), ncol=ncol(index))

comb.l5=matrix(l5[index,4],nrow=nrow(index), ncol=ncol(index))

comb.l6=matrix(l6[index,4],nrow=nrow(index), ncol=ncol(index))

sd.l0=apply(comb.l0,2,sd,na.rm=TRUE)

sd.l1=apply(comb.l1,2,sd,na.rm=TRUE)

sd.l2=apply(comb.l2,2,sd,na.rm=TRUE)

sd.l3=apply(comb.l3,2,sd,na.rm=TRUE)

sd.l4=apply(comb.l4,2,sd,na.rm=TRUE)

sd.l5=apply(comb.l5,2,sd,na.rm=TRUE)

sd.l6=apply(comb.l6,2,sd,na.rm=TRUE)

sd.l=rbind(sd.l0,sd.l1,sd.l2,sd.l3,sd.l4,sd.l5,sd.l6)

wt.df=rep(wt,ncol(sd.l))

wt.df=matrix(wt.df,nrow=length(absc[,1]))

wt.df=as.data.frame(wt.df)

wtsd.l=wt.df*sd.l

rm(comb.l0)

rm(comb.l1)

rm(comb.l2)

rm(comb.l3)

rm(comb.l4)

rm(comb.l5)

rm(comb.l6)

rm(sd.l0)

rm(sd.l1)

rm(sd.l2)

rm(sd.l3)

rm(sd.l4)

rm(sd.l5)

rm(sd.l6)

sumwt=sum(wt)

sumx=sum(wt*absc[,1])

sumy=colSums(wtsd.l)

xsquare=sum(wt*absc[,1]^2)

ysquare=colSums(wtsd.l^2)

wtx=wt*absc[,1]

prod.sdl=absc[,1]*wtsd.l

sumprod.sdl=colSums(prod.sdl)

sl=(sumwt*sumprod.sdl-sumx*sumy)/(sumwt*xsquare-sumx^2)

int=(sumy-sl*sumx)/sumwt

meanxw=sumx/sumwt

meanyw=sumy/sumwt

meanyw=rep(meanyw,length(absc[,1]))

meanyw=matrix(meanyw,nrow=length(absc[,1]),byrow=T)

difx=wt*(absc[,1]-meanxw)

wtdifx=rep(difx,ncol(sd.l))

wtdifmat=matrix(wtdifx,nrow=length(absc[,1]))

wtdifmat=as.data.frame(wtdifmat)

dify=sd.l-meanyw

covxyw=colSums(wtdifmat*dify)/sumwt

covxxw=sum(difx*(absc[,1]-meanxw))/sumwt

covxxw=rep(covxxw,ncol(sd.l))

covyyw=colSums(wt*dify^2)/sumwt

rsq=covxyw^2/(covxxw*covyyw)

nni=length(subset(int,int>=0))/length(int)

**Script 10: combinatorial calculation of regression coefficients, Theil’s regression, 6 spiking levels**

print("Select files containing drug levels and assay results")

aepconc=read.csv(file.choose())

aepresults=read.csv(file.choose())

l0=subset(aepresults,aepresults$lvl==0)

l1=subset(aepresults,aepresults$lvl==1)

l2=subset(aepresults,aepresults$lvl==2)

l3=subset(aepresults,aepresults$lvl==3)

l4=subset(aepresults,aepresults$lvl==4)

l5=subset(aepresults,aepresults$lvl==5)

l6=subset(aepresults,aepresults$lvl==6)

sd.l0=NULL

sd.l1=NULL

sd.l2=NULL

sd.l3=NULL

sd.l4=NULL

sd.l5=NULL

sd.l6=NULL

int=NULL

sl=NULL

absc=aepconc[colnames(aepconc)==colnames(aepresults)[[4]]]

indep=absc[,1]

j=20

index=combn(nrow(l0),j)

for (i in 1:ncol(index)) {

sd.l0[i]=sd(subset(l0[l0$sample[index[,i]],4],l0[l0$sample[index[,i]],4]!="NA"))

sd.l1[i]=sd(subset(l1[l1$sample[index[,i]],4],l1[l1$sample[index[,i]],4]!="NA"))

sd.l2[i]=sd(subset(l2[l2$sample[index[,i]],4],l2[l2$sample[index[,i]],4]!="NA"))

sd.l3[i]=sd(subset(l3[l3$sample[index[,i]],4],l3[l3$sample[index[,i]],4]!="NA"))

sd.l4[i]=sd(subset(l4[l4$sample[index[,i]],4],l4[l4$sample[index[,i]],4]!="NA"))

sd.l5[i]=sd(subset(l5[l5$sample[index[,i]],4],l5[l5$sample[index[,i]],4]!="NA"))

sd.l6[i]=sd(subset(l6[l6$sample[index[,i]],4],l6[l6$sample[index[,i]],4]!="NA"))

ord=c(sd.l0[i],sd.l1[i],sd.l2[i],sd.l3[i],sd.l4[i],sd.l5[i],sd.l6[i])

reg=mblm(ord~indep, repeated=TRUE)

sl[i]=summary(reg)$coefficients[[2]]

int[i]=summary(reg)$coefficients[[1]]

}

nni=length(subset(int,int>=0))/length(int)

**Script 11: combinatorial calculation of regression coefficients, unweighted linear least squares regression, 10 spiking levels**

print("Select files containing drug levels and assay results")

aepconc=read.csv(file.choose())

aepresults=read.csv(file.choose())

l0=subset(aepresults,aepresults$lvl==0)

l1=subset(aepresults,aepresults$lvl==1)

l2=subset(aepresults,aepresults$lvl==2)

l3=subset(aepresults,aepresults$lvl==3)

l4=subset(aepresults,aepresults$lvl==4)

l5=subset(aepresults,aepresults$lvl==5)

l6=subset(aepresults,aepresults$lvl==6)

l7=subset(aepresults,aepresults$lvl==7)

l8=subset(aepresults,aepresults$lvl==8)

l9=subset(aepresults,aepresults$lvl==9)

l10=subset(aepresults,aepresults$lvl==10)

sd.l0=NULL

sd.l1=NULL

sd.l2=NULL

sd.l3=NULL

sd.l4=NULL

sd.l5=NULL

sd.l6=NULL

sd.l7=NULL

sd.l8=NULL

sd.l9=NULL

sd.l10=NULL

int=NULL

sl=NULL

rsq=NULL

absc=aepconc[colnames(aepconc)==colnames(aepresults)[[4]]]

j=20

index=combn(nrow(l0),j)

comb.l0=matrix(l0[index,4],nrow=nrow(index), ncol=ncol(index))

comb.l1=matrix(l1[index,4],nrow=nrow(index), ncol=ncol(index))

comb.l2=matrix(l2[index,4],nrow=nrow(index), ncol=ncol(index))

comb.l3=matrix(l3[index,4],nrow=nrow(index), ncol=ncol(index))

comb.l4=matrix(l4[index,4],nrow=nrow(index), ncol=ncol(index))

comb.l5=matrix(l5[index,4],nrow=nrow(index), ncol=ncol(index))

comb.l6=matrix(l6[index,4],nrow=nrow(index), ncol=ncol(index))

comb.l7=matrix(l7[index,4],nrow=nrow(index), ncol=ncol(index))

comb.l8=matrix(l8[index,4],nrow=nrow(index), ncol=ncol(index))

comb.l9=matrix(l9[index,4],nrow=nrow(index), ncol=ncol(index))

comb.l10=matrix(l10[index,4],nrow=nrow(index), ncol=ncol(index))

sd.l0=apply(comb.l0,2,sd,na.rm=TRUE)

sd.l1=apply(comb.l1,2,sd,na.rm=TRUE)

sd.l2=apply(comb.l2,2,sd,na.rm=TRUE)

sd.l3=apply(comb.l3,2,sd,na.rm=TRUE)

sd.l4=apply(comb.l4,2,sd,na.rm=TRUE)

sd.l5=apply(comb.l5,2,sd,na.rm=TRUE)

sd.l6=apply(comb.l6,2,sd,na.rm=TRUE)

sd.l7=apply(comb.l7,2,sd,na.rm=TRUE)

sd.l8=apply(comb.l8,2,sd,na.rm=TRUE)

sd.l9=apply(comb.l9,2,sd,na.rm=TRUE)

sd.l10=apply(comb.l10,2,sd,na.rm=TRUE)

sd.l=rbind(sd.l0,sd.l1,sd.l2,sd.l3,sd.l4,sd.l5,sd.l6,sd.l7,sd.l8,sd.l9,sd.l10)

rm(index)

rm(comb.l0)

rm(comb.l1)

rm(comb.l2)

rm(comb.l3)

rm(comb.l4)

rm(comb.l5)

rm(comb.l6)

rm(comb.l7)

rm(comb.l8)

rm(comb.l9)

rm(comb.l10)

rm(sd.l0)

rm(sd.l1)

rm(sd.l2)

rm(sd.l3)

rm(sd.l4)

rm(sd.l5)

rm(sd.l6)

rm(sd.l7)

rm(sd.l8)

rm(sd.l9)

rm(sd.l10)

sumy=colSums(sd.l)

ysquare=colSums(sd.l^2)

absc.df=rep(absc,ncol(sd.l))

absc.df=as.data.frame(absc.df)

prod.sdl=absc.df*sd.l

sumprod.sdl=colSums(prod.sdl)

sl=(nrow(absc)*sumprod.sdl-sum(absc[,1])*sumy)/(nrow(absc)*sum(absc^2)-(sum(absc))^2)

int=(sumy-sl*sum(absc))/nrow(absc)

rsd=apply(sd.l,2,sd)

sd.absc=sd(absc[,1])

sdx=rep(sd.absc,length(rsd))

sdmeans=colMeans(sd.l)

sdmeans.m=t(matrix(rep(sdmeans,nrow(sd.l)),ncol=nrow(sd.l)))

dify=sd.l-sdmeans.m

dify=as.data.frame(dify)

difx=absc-colMeans(absc)

difx.df=rep(difx,ncol(sd.l))

ncoldifx.df=as.data.frame(difx.df)

proddif=difx.df*dify

sdcov=colSums(proddif)/(nrow(absc)-1)

rsq=(sdcov/(sdx*rsd))^2

nni=length(subset(int,int>=0))/length(int)

**Script 12: combinatorial calculation of regression coefficients, unweighted 2^nd^-order least squares regression, 10 spiking levels**

print("Select files containing drug levels and assay results")

aepconc=read.csv(file.choose())

aepresults=read.csv(file.choose())

l0=subset(aepresults,aepresults$lvl==0)

l1=subset(aepresults,aepresults$lvl==1)

l2=subset(aepresults,aepresults$lvl==2)

l3=subset(aepresults,aepresults$lvl==3)

l4=subset(aepresults,aepresults$lvl==4)

l5=subset(aepresults,aepresults$lvl==5)

l6=subset(aepresults,aepresults$lvl==6)

l7=subset(aepresults,aepresults$lvl==7)

l8=subset(aepresults,aepresults$lvl==8)

l9=subset(aepresults,aepresults$lvl==9)

l10=subset(aepresults,aepresults$lvl==10)

sd.l0=NULL

sd.l1=NULL

sd.l2=NULL

sd.l3=NULL

sd.l4=NULL

sd.l5=NULL

sd.l6=NULL

sd.l7=NULL

sd.l8=NULL

sd.l9=NULL

sd.l10=NULL

int=NULL

sl1=NULL

sl2=NULL

rsq=NULL

absc=aepconc[colnames(aepconc)==colnames(aepresults)[[4]]]

indep=absc[,1]

j=6

index=combn(nrow(l0),j)

for (i in 1:ncol(index)) {

sd.l0[i]=sd(subset(l0[l0$sample[index[,i]],4],l0[l0$sample[index[,i]],4]!="NA"))

sd.l1[i]=sd(subset(l1[l1$sample[index[,i]],4],l1[l1$sample[index[,i]],4]!="NA"))

sd.l2[i]=sd(subset(l2[l2$sample[index[,i]],4],l2[l2$sample[index[,i]],4]!="NA"))

sd.l3[i]=sd(subset(l3[l3$sample[index[,i]],4],l3[l3$sample[index[,i]],4]!="NA"))

sd.l4[i]=sd(subset(l4[l4$sample[index[,i]],4],l4[l4$sample[index[,i]],4]!="NA"))

sd.l5[i]=sd(subset(l5[l5$sample[index[,i]],4],l5[l5$sample[index[,i]],4]!="NA"))

sd.l6[i]=sd(subset(l6[l6$sample[index[,i]],4],l6[l6$sample[index[,i]],4]!="NA"))

sd.l7[i]=sd(subset(l7[l7$sample[index[,i]],4],l7[l7$sample[index[,i]],4]!="NA"))

sd.l8[i]=sd(subset(l8[l8$sample[index[,i]],4],l8[l8$sample[index[,i]],4]!="NA"))

sd.l9[i]=sd(subset(l9[l9$sample[index[,i]],4],l9[l9$sample[index[,i]],4]!="NA"))

sd.l10[i]=sd(subset(l10[l10$sample[index[,i]],4],l10[l10$sample[index[,i]],4]!="NA"))

ord=c(sd.l0[i],sd.l1[i],sd.l2[i],sd.l3[i],sd.l4[i],sd.l5[i],sd.l6[i],sd.l7[i],sd.l8[i],sd.l9[i],sd.l10[i])

reg=lm(ord~poly(indep,2,raw=TRUE))

sl1[i]=reg$coefficients[[2]]

sl2[i]=reg$coefficients[[3]]

int[i]=reg$coefficients[[1]]

rsq[i]=summary(reg)$r.squared

}

nni=length(subset(int,int>=0))/length(int)

**Script 13: combinatorial calculation of regression coefficients, unweighted 3^rd^-order least squares regression, 10 spiking levels**

print("Select files containing drug levels and assay results")

aepconc=read.csv(file.choose())

aepresults=read.csv(file.choose())

l0=subset(aepresults,aepresults$lvl==0)

l1=subset(aepresults,aepresults$lvl==1)

l2=subset(aepresults,aepresults$lvl==2)

l3=subset(aepresults,aepresults$lvl==3)

l4=subset(aepresults,aepresults$lvl==4)

l5=subset(aepresults,aepresults$lvl==5)

l6=subset(aepresults,aepresults$lvl==6)

l6=subset(aepresults,aepresults$lvl==6)

l7=subset(aepresults,aepresults$lvl==7)

l8=subset(aepresults,aepresults$lvl==8)

l9=subset(aepresults,aepresults$lvl==9)

l10=subset(aepresults,aepresults$lvl==10)

sd.l0=NULL

sd.l1=NULL

sd.l2=NULL

sd.l3=NULL

sd.l4=NULL

sd.l5=NULL

sd.l6=NULL

sd.l7=NULL

sd.l8=NULL

sd.l9=NULL

sd.l10=NULL

int=NULL

sl1=NULL

sl2=NULL

sl3=NULL

rsq=NULL

absc=aepconc[colnames(aepconc)==colnames(aepresults)[[4]]]

indep=absc[,1]

j=6

index=combn(nrow(l0),j)

for (i in 1:ncol(index)) {

sd.l0[i]=sd(subset(l0[l0$sample[index[,i]],4],l0[l0$sample[index[,i]],4]!="NA"))

sd.l1[i]=sd(subset(l1[l1$sample[index[,i]],4],l1[l1$sample[index[,i]],4]!="NA"))

sd.l2[i]=sd(subset(l2[l2$sample[index[,i]],4],l2[l2$sample[index[,i]],4]!="NA"))

sd.l3[i]=sd(subset(l3[l3$sample[index[,i]],4],l3[l3$sample[index[,i]],4]!="NA"))

sd.l4[i]=sd(subset(l4[l4$sample[index[,i]],4],l4[l4$sample[index[,i]],4]!="NA"))

sd.l5[i]=sd(subset(l5[l5$sample[index[,i]],4],l5[l5$sample[index[,i]],4]!="NA"))

sd.l6[i]=sd(subset(l6[l6$sample[index[,i]],4],l6[l6$sample[index[,i]],4]!="NA"))

sd.l7[i]=sd(subset(l7[l7$sample[index[,i]],4],l7[l7$sample[index[,i]],4]!="NA"))

sd.l8[i]=sd(subset(l8[l8$sample[index[,i]],4],l8[l8$sample[index[,i]],4]!="NA"))

sd.l9[i]=sd(subset(l9[l9$sample[index[,i]],4],l9[l9$sample[index[,i]],4]!="NA"))

sd.l10[i]=sd(subset(l10[l10$sample[index[,i]],4],l10[l10$sample[index[,i]],4]!="NA"))

ord=c(sd.l0[i],sd.l1[i],sd.l2[i],sd.l3[i],sd.l4[i],sd.l5[i],sd.l6[i],sd.l7[i],sd.l8[i],sd.l9[i],sd.l10[i])

reg=lm(ord~poly(indep,3,raw=TRUE))

sl1[i]=reg$coefficients[[2]]

sl2[i]=reg$coefficients[[3]]

sl3[i]=reg$coefficients[[4]]

int[i]=reg$coefficients[[1]]

rsq[i]=summary(reg)$r.squared

}

nni=length(subset(int,int>=0))/length(int)

**Script 14: combinatorial calculation of regression coefficients, 1/x^2^-weighted linear least squares regression, 10 spiking levels**

print("Select files containing drug levels and assay results")

aepconc=read.csv(file.choose())

aepresults=read.csv(file.choose())

aepconc[1,2:ncol(aepconc)]=0.0000001

l0=subset(aepresults,aepresults$lvl==0)

l1=subset(aepresults,aepresults$lvl==1)

l2=subset(aepresults,aepresults$lvl==2)

l3=subset(aepresults,aepresults$lvl==3)

l4=subset(aepresults,aepresults$lvl==4)

l5=subset(aepresults,aepresults$lvl==5)

l6=subset(aepresults,aepresults$lvl==6)

l7=subset(aepresults,aepresults$lvl==7)

l8=subset(aepresults,aepresults$lvl==8)

l9=subset(aepresults,aepresults$lvl==9)

l10=subset(aepresults,aepresults$lvl==10)

sd.l0=NULL

sd.l1=NULL

sd.l2=NULL

sd.l3=NULL

sd.l4=NULL

sd.l5=NULL

sd.l6=NULL

int=NULL

sl=NULL

absc=aepconc[colnames(aepconc)==colnames(aepresults)[[4]]]

j=6

wt=1/absc[,1]^2

index=combn(nrow(l0),j)

comb.l0=matrix(l0[index,4],nrow=nrow(index), ncol=ncol(index))

comb.l1=matrix(l1[index,4],nrow=nrow(index), ncol=ncol(index))

comb.l2=matrix(l2[index,4],nrow=nrow(index), ncol=ncol(index))

comb.l3=matrix(l3[index,4],nrow=nrow(index), ncol=ncol(index))

comb.l4=matrix(l4[index,4],nrow=nrow(index), ncol=ncol(index))

comb.l5=matrix(l5[index,4],nrow=nrow(index), ncol=ncol(index))

comb.l6=matrix(l6[index,4],nrow=nrow(index), ncol=ncol(index))

comb.l7=matrix(l7[index,4],nrow=nrow(index), ncol=ncol(index))

comb.l8=matrix(l8[index,4],nrow=nrow(index), ncol=ncol(index))

comb.l9=matrix(l9[index,4],nrow=nrow(index), ncol=ncol(index))

comb.l10=matrix(l10[index,4],nrow=nrow(index), ncol=ncol(index))

sd.l0=apply(comb.l0,2,sd,na.rm=TRUE)

sd.l1=apply(comb.l1,2,sd,na.rm=TRUE)

sd.l2=apply(comb.l2,2,sd,na.rm=TRUE)

sd.l3=apply(comb.l3,2,sd,na.rm=TRUE)

sd.l4=apply(comb.l4,2,sd,na.rm=TRUE)

sd.l5=apply(comb.l5,2,sd,na.rm=TRUE)

sd.l6=apply(comb.l6,2,sd,na.rm=TRUE)

sd.l7=apply(comb.l7,2,sd,na.rm=TRUE)

sd.l8=apply(comb.l8,2,sd,na.rm=TRUE)

sd.l9=apply(comb.l9,2,sd,na.rm=TRUE)

sd.l10=apply(comb.l10,2,sd,na.rm=TRUE)

sd.l=rbind(sd.l0,sd.l1,sd.l2,sd.l3,sd.l4,sd.l5,sd.l6,sd.l7,sd.l8,sd.l9,sd.l10)

wt.df=rep(wt,ncol(sd.l))

wt.df=matrix(wt.df,nrow=length(absc[,1]))

wt.df=as.data.frame(wt.df)

wtsd.l=wt.df*sd.l

rm(comb.l0)

rm(comb.l1)

rm(comb.l2)

rm(comb.l3)

rm(comb.l4)

rm(comb.l5)

rm(comb.l6)

rm(comb.l7)

rm(comb.l8)

rm(comb.l9)

rm(comb.l10)

rm(sd.l0)

rm(sd.l1)

rm(sd.l2)

rm(sd.l3)

rm(sd.l4)

rm(sd.l5)

rm(sd.l6)

rm(sd.l7)

rm(sd.l8)

rm(sd.l9)

rm(sd.l10)

sumwt=sum(wt)

sumx=sum(wt*absc[,1])

sumy=colSums(wtsd.l)

xsquare=sum(wt*absc[,1]^2)

ysquare=colSums(wtsd.l^2)

wtx=wt*absc[,1]

prod.sdl=absc[,1]*wtsd.l

sumprod.sdl=colSums(prod.sdl)

sl=(sumwt*sumprod.sdl-sumx*sumy)/(sumwt*xsquare-sumx^2)

int=(sumy-sl*sumx)/sumwt

meanxw=sumx/sumwt

meanyw=sumy/sumwt

meanyw=rep(meanyw,length(absc[,1]))

meanyw=matrix(meanyw,nrow=length(absc[,1]),byrow=T)

difx=wt*(absc[,1]-meanxw)

wtdifx=rep(difx,ncol(sd.l))

wtdifmat=matrix(wtdifx,nrow=length(absc[,1]))

wtdifmat=as.data.frame(wtdifmat)

dify=sd.l-meanyw

covxyw=colSums(wtdifmat*dify)/sumwt

covxxw=sum(difx*(absc[,1]-meanxw))/sumwt

covxxw=rep(covxxw,ncol(sd.l))

covyyw=colSums(wt*dify^2)/sumwt

rsq=covxyw^2/(covxxw*covyyw)

nni=length(subset(int,int>=0))/length(int)

**Script 15: combinatorial calculation of regression coefficients, Theil’s regression, 10 spiking levels**

print("Select files containing drug levels and assay results")

aepconc=read.csv(file.choose())

aepresults=read.csv(file.choose())

l0=subset(aepresults,aepresults$lvl==0)

l1=subset(aepresults,aepresults$lvl==1)

l2=subset(aepresults,aepresults$lvl==2)

l3=subset(aepresults,aepresults$lvl==3)

l4=subset(aepresults,aepresults$lvl==4)

l5=subset(aepresults,aepresults$lvl==5)

l6=subset(aepresults,aepresults$lvl==6)

l7=subset(aepresults,aepresults$lvl==7)

l8=subset(aepresults,aepresults$lvl==8)

l9=subset(aepresults,aepresults$lvl==9)

l10=subset(aepresults,aepresults$lvl==10)

sd.l0=NULL

sd.l1=NULL

sd.l2=NULL

sd.l3=NULL

sd.l4=NULL

sd.l5=NULL

sd.l6=NULL

sd.l7=NULL

sd.l8=NULL

sd.l9=NULL

sd.l10=NULL

int=NULL

sl=NULL

absc=aepconc[colnames(aepconc)==colnames(aepresults)[[4]]]

indep=absc[,1]

j=20

index=combn(nrow(l0),j)

for (i in 1:ncol(index)) {

sd.l0[i]=sd(subset(l0[l0$sample[index[,i]],4],l0[l0$sample[index[,i]],4]!="NA"))

sd.l1[i]=sd(subset(l1[l1$sample[index[,i]],4],l1[l1$sample[index[,i]],4]!="NA"))

sd.l2[i]=sd(subset(l2[l2$sample[index[,i]],4],l2[l2$sample[index[,i]],4]!="NA"))

sd.l3[i]=sd(subset(l3[l3$sample[index[,i]],4],l3[l3$sample[index[,i]],4]!="NA"))

sd.l4[i]=sd(subset(l4[l4$sample[index[,i]],4],l4[l4$sample[index[,i]],4]!="NA"))

sd.l5[i]=sd(subset(l5[l5$sample[index[,i]],4],l5[l5$sample[index[,i]],4]!="NA"))

sd.l6[i]=sd(subset(l6[l6$sample[index[,i]],4],l6[l6$sample[index[,i]],4]!="NA"))

sd.l7[i]=sd(subset(l7[l7$sample[index[,i]],4],l7[l7$sample[index[,i]],4]!="NA"))

sd.l8[i]=sd(subset(l8[l8$sample[index[,i]],4],l8[l8$sample[index[,i]],4]!="NA"))

sd.l9[i]=sd(subset(l9[l9$sample[index[,i]],4],l9[l9$sample[index[,i]],4]!="NA"))

sd.l10[i]=sd(subset(l10[l10$sample[index[,i]],4],l10[l10$sample[index[,i]],4]!="NA"))

ord=c(sd.l0[i],sd.l1[i],sd.l2[i],sd.l3[i],sd.l4[i],sd.l5[i],sd.l6[i],sd.l7[i],sd.l8[i],sd.l9[i],sd.l10[i])

reg=mblm(ord~indep, repeated=FALSE)

sl[i]=summary(reg)$coefficients[[2]]

int[i]=summary(reg)$coefficients[[1]]

}

nni=length(subset(int,int>=0))/length(int)

**Script 16: Regression on the final precision profiles (N=24, M=20+blank) using algorithms (a)-(f)**

regdata=read.csv(file.choose())

x=regdata[,1]

x[[1]]=x[[1]]+0.0000001

y=regdata[,2]

wt=1/(x^2)

head=c("concentration","sd","ts","siegel","wls","ols","2-poly","3-poly")

olsout=lm(y~x)

wlsout=lm(y~x, weights = wt)

tsout=mblm(y~x,repeated=FALSE)

tssout=mblm(y~x,repeated=TRUE)

olsy=as.data.frame(predict(olsout,data.frame(x,y),interval='confidence',level=0.99))$fit

olsrsq=summary(lm(y~x))$r.squared

tsy=tsout$coefficients[[1]]+tsout$coefficients[[2]]*x

tssy=tssout$coefficients[[1]]+tsout$coefficients[[2]]*x

wlsy=as.data.frame(predict(wlsout,data.frame(x,y),interval='confidence',level=0.99))$fit

wlsrsq=summary(wlsout)$r.squared

polyfit=lm(y~poly(x,2,raw=TRUE))

polyrsq=summary(lm(y~poly(x,2)))$r.squared

predi=as.data.frame(predict(polyfit,data.frame(x,y),interval='confidence',level=0.99))$fit

predi=as.data.frame(cbind(x,y,predi))

polyfit.3rd=lm(y~poly(x,3,raw=TRUE))

polyrsq.3rd=summary(lm(y~poly(x,3)))$r.squared

predi.3rd=as.data.frame(predict(polyfit.3rd,data.frame(x,y),interval='confidence',level=0.99))$fit

predi.3rd=as.data.frame(cbind(x,y,predi.3rd))

rsd=100*y/x

outdata=data.frame(x,y,tsy,tssy,wlsy,olsy,predi[,3],predi.3rd[,3],rsd)

outdata.perc=100*outdata/y

outdata.perc=outdata.perc[,-9]

outdata.perc[,1:2]=outdata[,1:2]

colnames(outdata.perc)=c("concentration","sd","ts","siegel","wls","ols","2-poly","3-poly")

colnames(outdata)=c("concentration","sd","ts","siegel","wls","ols","2-poly","3-poly","rsd")

ggplot(regdata,aes(conc,sd))+geom_point(size=6)+

geom_segment(aes(x =0, y=tsy[1], xend=regdata$conc[21], yend=tsy[21]), color="yellow", size=1.5)+

geom_segment(aes(x =0, y=tssy[1], xend=regdata$conc[21], yend=tssy[21]), color="darkgreen", size=1.5)+

geom_segment(aes(x =0, y=wlsy[1], xend=regdata$conc[21], yend=wlsy[21]), color="blue", size=1.5)+

geom_segment(aes(x =0, y=olsy[1], xend=regdata$conc[21], yend=olsy[21]), color="black", size=1.5)+

geom_smooth(method="lm",formula=y~poly(x,2,raw=TRUE),se=F,color="red",size=1.5)+

geom_smooth(method="lm",formula=y~poly(x,3,raw=TRUE),se=F,color="orange", size=1.5)+

labs(x="concentration (mg/L)",y="standard deviation (mg/L)")+

scale_x_continuous(limit=c(-0.2,190),breaks=seq(from=0,to=160,by=20))+

scale_y_continuous(limit=c(-0.2,10),breaks=seq(from=0,to=10,by=1))+

theme(axis.text.x = element_text(size=32),axis.text.y.left = element_text(size=32))+

theme(axis.title.x = element_text(size=32),axis.title.y = element_text(size=32))
